# Preparation and Characterization of a Series of Self-Healable Bio-Based Poly(thiourethane) Vitrimer-like Materials

**DOI:** 10.3390/polym15061583

**Published:** 2023-03-22

**Authors:** Federico Guerrero, Xavier Ramis, Silvia De la Flor, Àngels Serra

**Affiliations:** 1 Analytical and Organic Chemistry Department, Universitat Rovira i Virgili, C/ Marcel·lí Domingo s/n Edificio N4, 43007 Tarragona, Spain; 2 Thermodynamics Laboratory, Universitat Politècnica de Catalunya ETSEIB, Av. Diagonal 647, 08028 Barcelona, Spain; 3 Department of Mechanical Engineering, Universitat Rovira i Virgili, Av. Països Catalans, 26, 43007 Tarragona, Spain

**Keywords:** poly(thiourethanes), thiol, vitrimers, renewable, bio-based monomers

## Abstract

A series of poly(thiourethanes) (PTUs) from biobased monomers have been synthesized. Limonene and squalene were transformed into polyfunctional thiols by thiol-ene reaction with thioacetic acid and further saponification. They were then reacted in different proportions with hexamethylene diisocyanate (HDI) in the presence of a catalyst to prepare bio-based poly(thiourethane) vitrimer-like materials. The different functionalities of squalene and limonene thiols (six and two, respectively) allow for changing the characteristics of the final material by only varying their relative proportions in the reactive mixture. The proportions of thiol and isocyanate groups were stoichiometric in all the formulations tested. An acidic and a basic catalyst were tested in the preparation of the networked polymers. As the acidic catalyst, we selected dibutyltin dilaurate (DBTDL), and as the basic catalyst, a tetraphenylborate salt of 1,8-diazabicyclo(5.4.0)undec-7-ene (BGDBU), which has the advantage of only releasing the base at high temperatures. The materials obtained were characterized by thermogravimetry and thermomechanical analysis. The vitrimeric-like behavior was evaluated, and we could see that higher proportions of the limonene derivative in the formulations led to faster stress relaxation of the material. The use of the base catalyst led to a much shorter relaxation time. The materials obtained demonstrated good self-healing efficiency.

## 1. Introduction

On our planet, which is scarce in natural resources and whose population growth has aggravated, the depletion of fossil reserves is one of the main concerns. This, added to the harmful effects caused by their extraction, processing, and use, as well as to environmental legislation that responds to a society that is increasingly aware of it, has led to the search for more renewable and sustainable sources of organic compounds.

In this search, biomass is essential as a renewable source with a neutral carbon balance. One of the industries most dependent on fossil fuels is the polymer industry. For this reason, polymers derived from biomass are being widely developed. Among the different kinds of biomass, vegetable oils, carbohydrates, lignin, terpenes, and terpenoids [1,2] stand out. In the case of terpenes, their structural diversity and the presence of double bonds with different reactivities have made them building blocks with great potential in biopolymer production [3].

Another environmental concern our present civilization face is the elimination or recycling of thermosets. Although indispensable because of their outstanding mechanical and thermal characteristics, they lack degradability and, therefore, permanently fill landfills, constituting an enormous problem. To solve this issue, the inclusion of reversible groups in the network structure has been proposed. These materials are called covalent adaptable networks (CANs) or vitrimers and were proposed for the first time by Leibler’s group [4].

This new type of polymeric materials has been gaining attention thanks to its thermosetting characteristics but with the capability of being reshaped, reprocessed, and recycled. These re-processable polymer networks are cross-linked polymers with sufficient dynamic bonds for network reconfiguration under appropriate conditions. The topological rearrangement can be triggered by the application of stress above a specific temperature (called Topological Freezing Temperature, *T_v_*) and usually in the presence of a catalyst, leading to a relaxed stress state where the material is completely malleable at adequately high temperature but having a “vitrified” cross-linked topology at sufficiently low temperature [5].

The distinctive feature of associative CANs is the constant crosslinking density during the exchange reactions, implying no loss of material integrity. On the contrary, they exhibit a gradual decrease in viscosity when increasing temperature that follows the Arrhenius law for high temperature. This characteristic makes them very attractive from a technological point of view since it enables them to be processed over a wide range of temperatures, recycled, reshaped, self-healed, and self-welded, with potential use in the industrial field as lenses, coatings, adhesives, etc.

An outstanding contribution to sustainability in the thermosets field can be implemented using bio-based monomers to prepare covalent adaptable networks. By combining both strategies, we can effectively move toward a green chemistry model and a circular economy that would contribute to reaching the most significant challenge humankind has faced: net-zero carbon emission. There are some contributions to this topic recently reviewed by Avéroux et al. [6].

Our group has deeply studied the vitrimer-like characteristics of poly(thiourethane) (PTU) networks [7,8,9,10,11,12]. These materials have excellent optical properties and can easily be reshaped and recycled [13,14]. They are prepared through a click-type reaction of isocyanate monomers with thiol compounds in the presence of acid or basic catalysts. Although the use of isocyanates is not recommended due to their preparation from phosgene and their toxicity, there is no safer alternative in the preparation of PTUs as it occurs in their oxygen analogs, poly(urethane)s. Poly(urethane)s were firstly prepared by polycondensation of isocyanates and alcohols, but nowadays, there is a greener alternative based on cyclic carbonates [15]. The non-isocyanate polyurethanes (NIPUs) are prepared by the attack of nucleophilic amines on cyclic carbonates. A parallel procedure, based on the aminolysis of cyclodithiocarbonates led to the NIPTUs, with poly(mercapto thiourethane/polyether structures, with an interchanged position of O and S in comparison to conventional poly(thiourethanes) [16].

In the last decade, thiols from renewable sources have been employed in the preparation of biopolymers instead of petroleum-based thiols. The most used is cysteine, which is a semi-essential amino acid [17]. However, there are only a few, and it is more common to synthesize them from natural resources by several thiolation methods.

Isosorbide was converted into the corresponding dithiol by reaction with 3-mercaptopropionic acid, and then, it was photopolymerized by a thiol-ene procedure with neat tung or hazelnut oils [18].

Another procedure used to obtain thiols from renewable resources was the thiol-ene photochemical reaction of ene-compounds with thioacetic acid, followed by the saponification of the thioester formed. Acosta et al. [19] reported the preparation of hexathiolated squalene using this synthetic approximation. This squalene derivative (SQ6SH) was added to vinyl ether, acryl, and allyl monomers, which photochemically reacted by ene-homopolymerization and thiol-ene reaction. The same compound was polymerized with acrylated epoxidized soybean oil by Grauzeliene et al. [20] and with cycloaliphatic resins by Guzman et al. [21]. The presence of double bonds in limonene and β-pinene allowed their transformation into dithiols, which were polycondensed by Firdaus et al. [22] Poly(thiourethane) thermosetting coatings were prepared from polyfunctional bio-based thiols obtained by thiol-ene reaction of sucrose soya ester and limonene and several commercially available isocyanate trimers [23].

Limonene is naturally produced by a wide variety of plants, mainly citrus, despite the fact that it can also be obtained from the isomerization of pinene. It is a chiral molecule, with the (R)-enantiomer being the most abundant in nature, and its primary use is in the flavor and fragrance industry [2]. Squalene is naturally synthesized in plants, animals, bacteria, algae, and fungi as a precursor for synthesizing sterols, hormones, and vitamins. It can be extracted from the liver oil of sharks and from plants, with amaranth having the highest concentration of this triterpene. Olives are also a good source. Moreover, microorganisms have great potential to become the preferred source for squalene production by optimizing fermentation and using metabolic engineering. Its primary use is in the pharmaceutic industry due to its properties as an anticancer, antioxidant, detoxifier, skin hydrating, drug carrier, and emollients [22].

In the present work, a mixture of thiols derived from terpenes (squalene and limonene), prepared according to the procedure reported by Acosta et al. [19], has been reacted with hexamethylene diisocyanate (HDI). A typical acidic catalyst (dibutyltin dilaurate, DBTDL) has been used. As a greener catalytic alternative, a precursor of the basic catalyst, the tetraphenyl borate salt of 1,8-diazabiciclo[5.4.0]undec-7-ene (BGDBU), which was proposed by us in a previous study, has also been tested [9], and the resulting networked poly(thiourethanes) have been characterized and compared. Both catalysts enhance the trans-thiocarbamoylation reaction, which is responsible for the vitrimeric-like behavior.

The materials obtained were characterized by thermogravimetry and thermomechanical analysis. The vitrimeric-like behavior was evaluated, and we could see that higher proportions of the limonene derivative in the formulations leads to faster stress relaxation of the material. The use of the base catalyst led to a much shorter relaxation time. The materials obtained demonstrated good self-healing efficiency. In the present work, we have compared the materials with the different thiol compositions using DBTDL and BGDBU as catalysts to know the differences in the material’s characteristics and their relaxation behavior.

## 2. Materials and Methods

### 2.1. Materials

(R)-(+)-limonene (LM), hexamethylene diisocyanate (HDI), dibutyltin dilaurate (DBTDL), and sodium tetraphenylborate (NaBPh_4_) were obtained from Sigma Aldrich (Saint Louis, MO, USA). Squalene (SQ) and 1,8-diazabicyclo(5.4.0)undec-7-ene (DBU) were obtained from Alfa Aesar (Thermo Fisher, Kandel, Germany). Thioacetic acid (TAA) and 2,2-dimethoxy-2-phenylacetophenone (DMPA) were obtained from Acros Organics (Thermo Fisher Scientific, Geel, Belgium). Anhydrous magnesium sulfate (MgSO_4_) was obtained from (Thermo Fisher Scientific, Geel, Belgium). Sodium hydroxide (NaOH), hydrochloric acid (HCl), chloroform (CHCl_3_), and methanol (MeOH) were obtained from Scharlab (Barcelona, Spain). All products were used as received. BGDBU, which is a base generator, was synthesized from DBU and sodium tetraphenyl borate according to a reported methodology [24].

### 2.2. Synthesis of Renewable Thiols

Renewable thiols (SQ-S6 and LM-S2) were prepared by a two-step procedure according to the reported methodology [19]. The synthetic procedure started with the corresponding terpene (SQ or LM) that was irradiated at 356 nm with an excess of thioacetic acid (TAA) in the presence of DMPA. The corresponding thioacetate was saponified with a methanol solution of NaOH. Then, the organic phase was washed twice with distilled water and dried with MgSO_4_. The organic solvent was eliminated under vacuum. Yields were 94% (SQ-S6) and 90% (LM-S2). The products were characterized by ^1^H-NMR spectroscopy, and compared with those described [19,22].

### 2.3. Preparation of the Materials

Several formulations were prepared by mixing stoichiometric amounts of hexamethylene diisocyanate and the mixture of both thiols. Three different mixtures of thiols (SQ-S6 and LM-S2) were used (25/75, 50/50 and 75/25). Two different catalysts were tested with all formulations: an acidic catalyst, dibutyltin dilaurate (DBTDL), and a basic latent catalyst, 1,8-diazabicyclo(5.4.0)undec-7-ene tetraphenylborate (BGDBU). The amount of catalyst was 4% mol by mol of HDI in both cases. The nomenclature adopted for formulations and samples was SQLM XXYY Z, where XX was the weight percentage of SQ-S6 in reference to the total amount of thiol, YY was the percentage of LM-S2, and Z was the type of catalyst: acid or base. The composition of the formulations is shown in Table 1.

The formulations were prepared by mixing the components at 80 °C and placing them on Petri dishes covered with adhesive Teflon to avoid sticking to the glass. The mixtures were then heated at 140 °C for 30 min. Then, the flexible materials were removed from the mold, and the curing process was completed in a hot press at 190 °C for 90 min under a pressure of 15 MPa. The cured samples were cut with heat to obtain rectangular specimens of about 20 × 5 × 0.5 mm.

### 2.4. Characterization Techniques

^1^H NMR spectra were recorded on a Varian VNMR-S400 NMR spectrometer (Varian, Lake Forest, CA, USA), using CDCl_3_ as the solvent. All chemical shifts are given in parts per million (ppm) on the δ scale, using the signal of undeuterated solvent as an internal standard (^1^H NMR: CDCl_3_ = 7.26 ppm).

A spectrometer FT/IR 6700 (Jasco, Hachioji, Tokyo, Japan), equipped with a Golden Gate ATR accessory (Specac-Tecknokroma, Barcelona, Spain), was used to record FTIR spectra. An interval from 650 to 4000 cm^−1^ was explored with a resolution of 4 cm^−1^, and 32 scans were performed for each spectrum. All spectra were recorded at room temperature.

Raman spectra were recorded with a Raman Renishaw InVia spectrometer (Gloucestershire, UK), with a resolution of 1 cm^−1^ in the spectral range from 400 to 3100 cm^−1^. The device was equipped with a heating plate accessory (Linkam, Salfords, UK), allowing the recording of spectra from room temperature to 200 °C.

A TGA 2 STAR System thermobalance (Mettler, Columbus, OH, USA) was used to evaluate the thermal stability of the materials. The experiments were performed under nitrogen (flow 50 mL/min). Samples of 10-15 mg of cured materials were degraded between 30 and 700 °C at a heating rate of 10 °C/min.

A DMA Q800 analyzer from (TA Instruments, New Castle, DE, USA) equipped with a film tension clamp, was employed to determine the viscoelastic and thermomechanical properties of the materials. The variation of tan *δ* and storage modulus on changing the temperature was investigated in the virgin materials and after several stress relaxations tests. The specimens were tested at a heating rate of 3 °C/min from 0 to 200 °C, with a frequency of 1 Hz and 0.05% strain. Tensile stress relaxation tests were performed with the same film tension clamp, tracking samples with the same dimensions previously defined. The samples were equilibrated at 175 °C and maintained at this temperature for 5 min. After that, a constant strain of 1.5% (which ensures the materials are in the linear range) was applied, and the stress level was measured during 20 min (for acid-catalyzed samples) and 5 min (for basic-catalyzed samples). Finally, the temperature was increased by 5 °C, and the process was iteratively repeated until the final temperature of 195 °C. The relaxation stress *σ*(*t*) was normalized by the initial stress *σ_o_*. The relaxation time *τ* was taken as the time needed to relax the 0.37*σ_o_*.

The activation energies of the relaxation processes, *E_a_*, were calculated using an Arrhenius-type equation, which takes the relaxation times obtained at each temperature:

(1) $$\ln\left(\tau \right)=\frac{E_{a}}{RT}-\ln A$$

where *τ* is the time needed to reach a given stress-relaxation value (0.37*σ_o_*), *A* is the pre-exponential factor, and *R* is the gas constant. The topology freezing temperature (*T_v_*) was obtained from the Arrhenius relation. *T_v_* is the temperature at which the material reaches a viscosity of 10^12^ Pa·s. By applying Maxwell’s relation and *E*’ obtained from DMA measurements (assuming that *E*’ is relatively invariant in the rubbery state), τ* was determined for each sample. The Arrhenius relationship was extrapolated to the corresponding value of τ* to determine *T_v_* for each material.

Creep and recovery properties were studied in tension with the DMA Q800 analyzer (TA Instruments, New Castle, DE, USA) using the film tension clamp. The SQLM 2575 ACID specimen was stretched under a stress of 0.1 MPa at 180 °C for 30 min. Then, the stress was immediately released, and the specimen was left to recover for 30 min. For comparative purposes between the rubbery and the vitrimeric state, the specimen was tested under the same creep conditions at 100 °C (slightly above *T_g_*).

To determine the viscosity at each temperature needed to represent the Angell Fragility Plot, a series of creep experiments were performed on films, taking temperatures between 145 and 195 °C, with an increase of 5 °C in each test. To perform the essays, the selected temperature was maintained for 3 min, and a stress level of 0.1 MPa was applied for 30 min. The viscosity *η* (Pa·s) was obtained from the creep curves, taking into account the linear part of the variation of the strain and fitting it with linear regression. The strain rate $\dot{\varepsilon }$ was obtained from the slope of the linear fit. The viscosity *η* was calculated according to the following equation:

(2) $$\eta =\frac{\sigma }{\dot{\varepsilon }}\;$$

and represented against *T_g_*/*T*, obtaining the Angell fragility plot.

Self-healing tests were made by scratching the specimens with a doctor blade. Then, they were maintained in an oven at 190 °C for a certain period of time and were inspected from time to time to monitor the evolution of the scratch by taking photographs using a Digital Microscope Leica DMS1000 (Wetzler, Germany).

## 3. Results and Discussion

### 3.1. Preparation of the Materials

**Figure 1:**
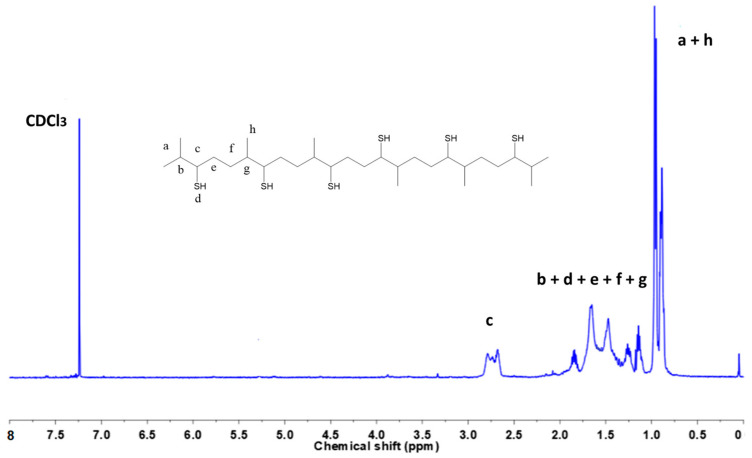
^1^H-NMR spectrum of the hexathiol derived from squalene in CDCl_3_.

**Figure 2:**
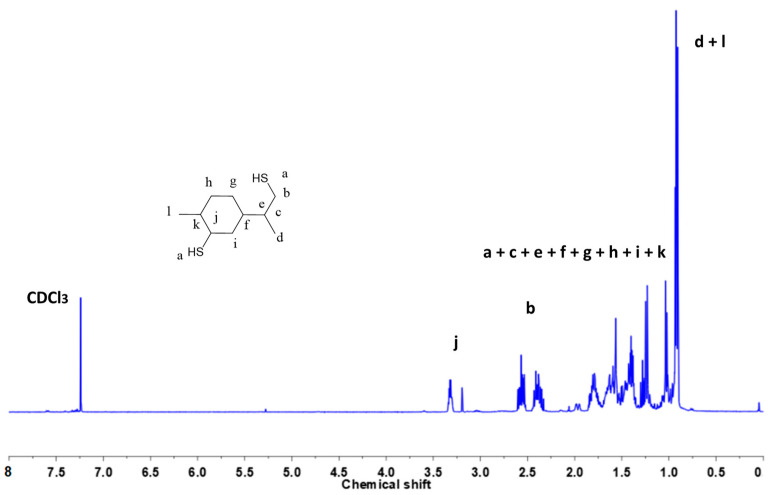
^1^H-NMR spectrum of the dithiol derived from limonene in CDCl_3_.

Firstly, thiols derived from squalene (SQ) and limonene (LM) were prepared using a photoinitiated thiol-ene reaction with thioacetic acid. This was followed by saponification of the thioester formed with NaOH, as described in the experimental section. The ^1^H NMR spectra of the thiols are represented in Figure 1 and Figure 2. As we can see, the spectra are quite complex due to the presence of stereoisomers and a large number of inequivalent protons in both molecules. However, the total absence of vinylic protons indicates that the thiol-ene reaction was completed. The spectra are similar to those previously reported for the squalene and limonene derivatives [19,22].

Once the bio-based thiols were obtained, thermosetting PTUs with different characteristics were produced by keeping the selected reactive mixture in the oven for the complete curing schedule, as explained in the experimental section. The curing schedule was optimized to reach the highest *T_g_* in each material.

**Scheme 1:**
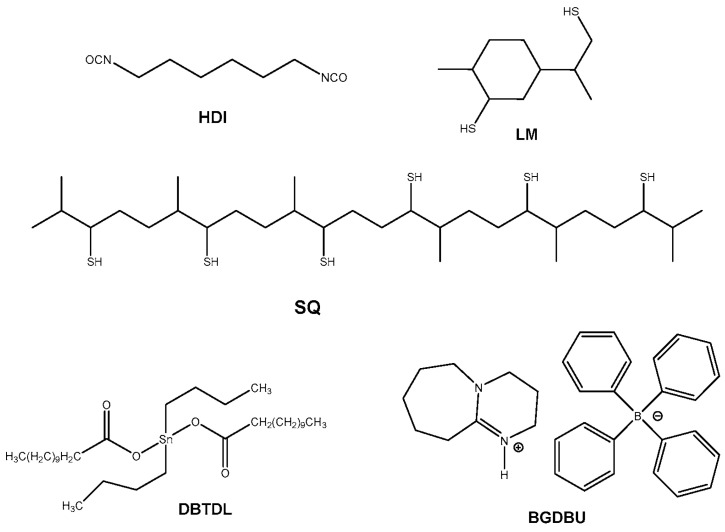
Structure of the starting monomers and catalysts.

Since limonene thiol (LM) has a functionality of two when reacting with isocyanates and the functionality of HDI is two, some proportion of hexathiolated squalene (SQ) is always required to obtain a tridimensional networked structure. Therefore, three formulations with different squalene/limonene thiol ratios have been studied. Because of its higher functionality, the higher the proportion of SQ in the formulation, the higher the crosslinking density. The high fragility of the material obtained from HDI and SQ without LM prevented further study of the material with the highest cross-linking density, and therefore, this formulation was not included in the study. Scheme 1 shows the structure of the monomers used in the preparation of the materials and the catalysts.

**Scheme 2:**
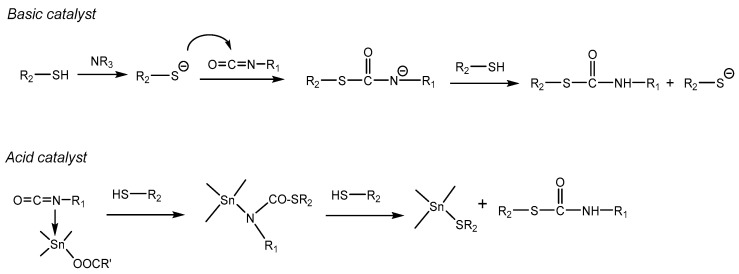
Proposed mechanism for the preparation of thiourethane groups in basic and acid conditions.

It is known that the reaction between isocyanates and thiols requires a catalyst. The most used are DBTDL [13] or a tertiary amine, such as trimethylamine or DBU [25]. Scheme 2 depicts the reported mechanisms of poly(thiourethane) formation in both basic and acidic conditions.

Whereas the use of amines transforms the thiol into thiolate, enhancing its nucleophilicity, the use of the tin compound increases the electrophilicity of the carbonyl group. It was reported that the isocyanate-thiol reaction initiated by a base has a click character, which assures that no secondary reactions occur.

To accelerate the stress relaxation process, the amount of catalyst in the material can be increased, but this leads to a dramatic increase in the curing rate when using a base, which makes it difficult to prepare reliable samples. For this reason, we have used a tetraphenyl borate salt (BGDBU) that, at a specific temperature, releases DBU, which acts as the basic catalyst [9,26]. This type of amidinium salt is an organocatalysts and presents latency since the curing does not start at room temperature but at temperatures higher than 100 °C. This fact allows preparing highly homogeneous PTUs in a straightforward procedure, with good temporal control of the curing process. The use of this organocatalyst does not requires any solvents. Because of the polar character of the solid BGDBU, its solubility in the reactive mixture is limited, and therefore a 4% mol by mol of HDI has been used, although we know that the higher the proportion of amine in the final material, the higher the relaxation rate [10]. The preparation of the materials was performed as explained in the experimental part.

### 3.2. Thermogravimetric Characterization of the Materials

**Figure 3:**
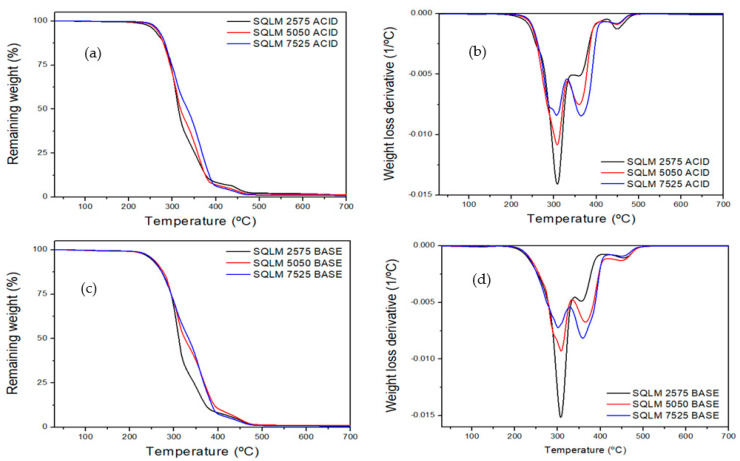
TGA and DTG curves of the materials prepared with acidic (**a**,**b**) and basic catalysts (**c**,**d**).

The thermal stability of the materials was evaluated by thermogravimetry. Figure 3 shows the weight loss curves and their first derivatives for all the materials prepared. The most interesting data are collected in Table 2.

In this table, we can see that the samples obtained with DBTDL are slightly more resistant to temperature than those obtained with the basic catalyst, but the differences are not significant. The composition of the material affects stability, as expected, since the higher the proportion of SQ in the formulation, the higher the initial degradation temperature due to the higher cross-linking density. There is also a slight influence of the composition in the final residue, which slightly increases with the cross-linking density. It is essential to highlight that the initial temperature of degradation is critical in the recycling process, as these materials can be safely reshaped at temperatures below 200 °C.

As shown in the DTG curves, the degradation occurs in two main steps for all materials without any difference when DBTDL or BGDBU were used as the catalysts. The derivative curves show that the higher the proportion of limonene moieties in the sample, the faster the first degradation step due to its lower functionality. In contrast, when the amount of the squalene derivative increases in the sample, the second degradation mechanism becomes faster, indicating the participation of the squalene network units in this process. Both peaks can be related to the reversion of thiourethane groups with the formation of isocyanates and thiols. However, they can also include the β-elimination of the ester groups in the remaining polythiourethane structures. The weak peak at higher temperatures corresponds to the breakage of the remaining bonds [9]. The character of the catalyst does not influence the degradation mechanism.

### 3.3. Thermodynamic Characterization of the Materials

**Figure 4:**
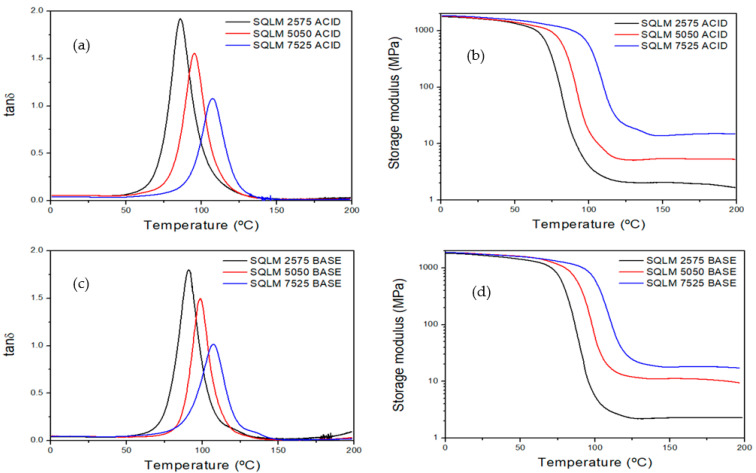
Evolution of tan *δ* and storage modulus with the temperature of the different materials prepared with acid (**a**,**b**) and basic catalysts (**c**,**d**).

Dynamic mechanical thermal analysis (DMTA) has been performed to evaluate the thermomechanical properties of the materials prepared and the influence of the proportion of both renewable thiols and the nature of the catalyst. The tan *δ* curves of all materials prepared are shown in Figure 4, and the main data extracted from the DMA analysis are collected in Table 3.

As can be foreseen, increasing the proportion of the thiol of squalene in the formulation results in a higher temperature of the maximum of tan *δ* due to the tighter network structure. Similarly, the moduli increases in the glassy and rubbery states. Although the limonene thiol has a functionality of two and therefore does not contribute to increasing the cross-linking density like squalene does, its rigid structure does not lead to a significant reduction in the tan d temperature when its proportion in the formulation is high. The increase in squalene derivative also increases the width of the tan δ curve, which reduces the homogeneity of the network structure and the damping capability. The use of the basic catalyst leads, in general, to a slightly higher tan *δ* temperature and moduli, but the differences are not significant.

If we compare these PTU samples to others previously developed by our research group, the present materials possess higher values of *T*_tan *δ*_ than the previous ones in which HDI was cross-linked with S4 with a base generator (*T*_tan *δ*_ = 77 °C) or with DBTDL (*T*_tan *δ*_ = 75 °C) [9].

### 3.4. Study of the Relaxation Process

In previous studies, our group demonstrated the vitrimer-like characteristics of poly(thiourethane) networks [7,8,10,11,12]. The interchange reaction responsible for the vitrimer-like behavior is the transthiocarbamoylation reaction. This mechanism involves the decomposition of thiourethane group to isocyanate and thiol, which instantly react to form thiourethane groups again. Thus, it is a dissociative mechanism but with a relaxation behavior typical of vitrimers involving an Arrhenius-type decrease in viscosity as if the degree of cross-linking remained unchanged. Therefore, PTUs can be qualified as vitrimeric-like materials.

**Figure 5:**
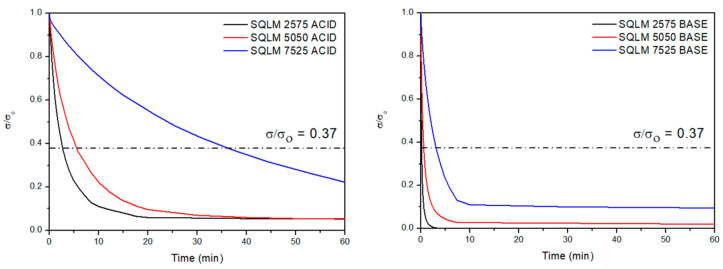
Normalized stress relaxation plots as a function of time for the different materials prepared with DBTDL (**left**) and with BGDBU (**right**) at 180 °C.

To determine the relaxation rate of these materials and analyze the effect of the proportion of both thiols and the kind of catalyst, DMTA stress relaxation tests at different temperatures from 175 to 195 °C were performed. Figure 5 shows the normalized stress relaxation curves of the prepared materials at 180 °C.

As observed in Figure 5, the materials with the lowest ratio of squalene (SQLM 2575) experience the fastest relaxation. Moreover, the materials prepared with a basic catalyst relax much faster than those prepared with DBTDL, as previously demonstrated [14]. The use of tetraphenylborate amidinium salts such as BGDBU allows for an increase in the amount of catalytic base without premature curing and reaching a faster relaxation. It has been demonstrated that the salt is even more efficient in the relaxation process than the free base [11]. This has been attributed to the presence of tetraphenyl boronic acid released during the activation of the base that acts as an additional catalyst. The values of the time to reach a relaxed stress state of *σ*/*σ_o_* = 0.37 (*τ*_0.37_) for all the materials are collected in Table 4.

As seen in Table 4, increasing the amount of squalene derivative in the mixture increases the *τ*_0.37_ with both catalysts. Moreover, materials containing BGDBU have shorter times than those catalyzed by DBTDL. In this table, it can be seen that the time to reach a *τ*_0.37_ is as short as 10 s for the SQLM 2575 sample catalyzed by BGDBU. This time of 10 s is the lowest time reached in all of our studies based on PTUs. Moreover, the material with the same monomer composition catalyzed by DBTDL also shows faster relaxation than previous materials catalyzed by this catalyst [7]. This is due to the higher proportion of catalysts in the present formulations. The salt BGDBU has limited solubility in the formulation due to its crystalline character. The studied formulations in the present study allow the solubilization of a higher amount of DGDBU; therefore, relaxation is more accelerated. Another fact that must be considered is the proportion of thiourethane groups in the network structure. In the present case, the proportion is higher than in previous materials prepared with trimethylolpropane tris(3-mercapto propionate) (S3) or tetrakis(3-mercaptopropionate) (S4). S3 and S4 have a higher thiol equivalent, leading to a lower proportion of reversible groups. Thus, the higher relaxation rate of the prepared materials, and their high vitreous transition temperatures, constitute an advantage of using the synthesized bio-based thiols.

**Figure 6:**
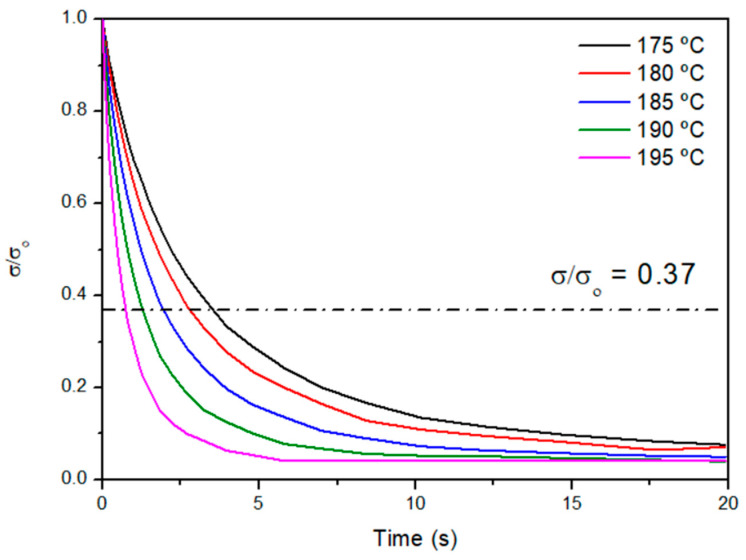
Normalized stress relaxation plots as a function of time at various temperatures from 175 to 195 °C for the sample SQLM 2575 catalyzed by DBTDL.

From the relaxation stress tests performed at different temperatures between 175 °C and 195 °C, the times to reach a relaxed stress state of *σ*/*σ_o_* = 0.37 (*τ*_0.37_) can be extracted, plotted versus 1000/T, and fitted to the Arrhenius relationship (Equation (1). From the Arrhenius equation and the relaxation times needed to attain a viscosity of 1012 Pa⋅s, *T_v_* (the topology freezing transition temperature) can also be calculated for each material. The main parameters of the Arrhenius equation are presented in Table 4, as well as their corresponding *T_v_*. Figure 6 presents, as an example, the variation of the relaxation of stresses on changing the temperature for the sample SQLM 2575 catalyzed by DBTDL. A similar tendency was observed in all the materials, with the relaxation time being lower at higher temperatures, as expected.

**Figure 7:**
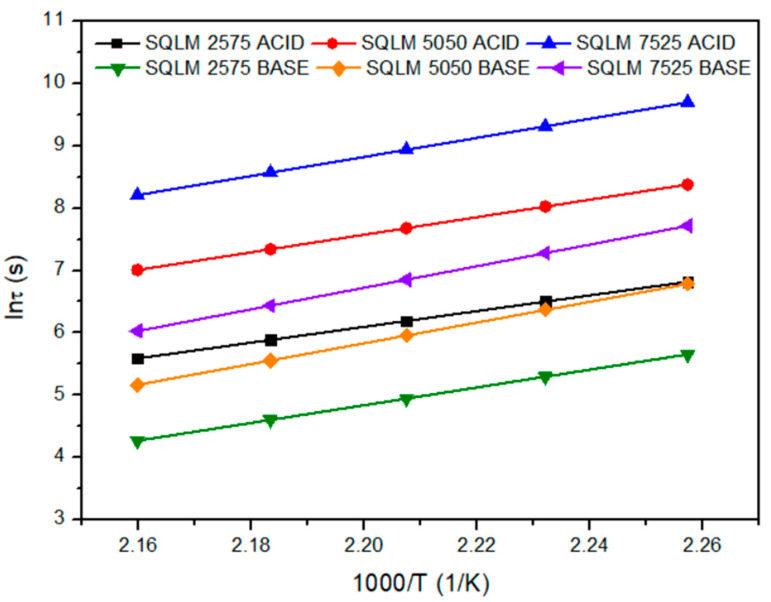
Arrhenius plot of relaxation times against temperature for all the materials prepared, measured from the stress relaxation experiments.

Figure 7 presents the Arrhenius plots deduced from the stress-relaxation tests for all the samples. As observed in the figure, the values obtained fit perfectly with an Arrhenius-like behavior, which confirms the previously reported vitrimer-like characteristics [7,10]. The activation energies calculated from the Arrhenius lines’ slope range from 105 to 144 kJ/mol, with higher values when DBTDL is the catalyst (see Table 4). As can be seen in Table 4, for all the materials, the *T_v_*s are higher than the corresponding *T_g_*s, which implies that the materials have to surpass both temperatures, not only the *T_g_*, to be reshapable. The *T_v_*s determined for the materials with both catalysts are similar, being slightly lower when DBTDL is used. Moreover, the higher the proportion of squalene, the higher the *T_v_*. The *T_v_*s calculated for the present materials are higher than those reported before [7,10], which were prepared from HDI and S3, and this can be attributed to the higher cross-linking density of the present ones.

**Figure 8:**
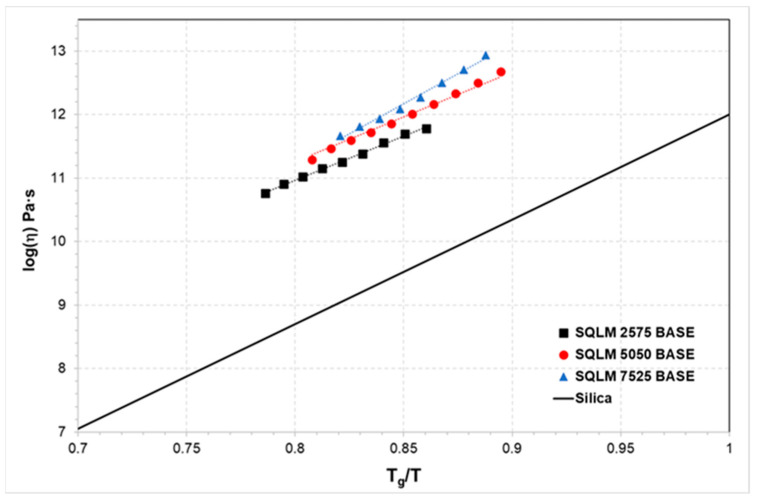
Angell fragility plot of the logarithm of the viscosity as a function of the inverse temperature, scaled to *T_g_* for the different materials catalyzed by BGDBU. For comparative purposes, the black continuous line, which is the relation for silica, has been included [27].

A series of creep experiments were performed to construct the Angell fragility plot. Figure 8 shows this plot for the samples catalyzed by BGDBU.

The fragility plot accounts for how rapidly the dynamics of a material slow down when cooled toward the topology freezing transition temperature (*T_v_*). Angell proposed the definition of fragility [27], which characterizes the viscosity slope against temperature. Among glass-forming liquids, silica has low fragility; therefore, it is considered a “strong glass former”. As seen in the figure, the viscosity dependence against temperature for our materials follows an Arrhenius law, as occurs in silica. Therefore, the PTUs prepared are “strong glass formers” compared to thermoplastics and dissociative CANs, which are “fragile liquids”.

From these plots, it can be deduced the activation energy and the *T_v_* for all the samples (results are presented in Table 5). It can be appreciated in Table 5 that both parameters, the activation energies and the topology freezing transition temperatures, are similar to those obtained from the relaxation tests and follow the same tendency, the highest activation energy and the highest *T_v_* are obtained in the sample with the highest proportion of squalene. The table also presents fragility indexes, which are defined as the rate of viscosity drop at *T_g_* and deduced from the Angell Fragility Plots.

**Figure 9:**
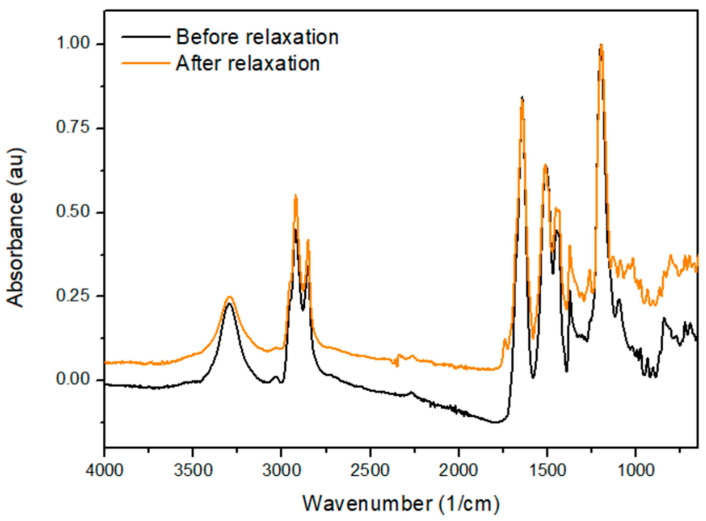
FTIR-ATR spectra of the sample SQLM 2575 catalyzed by DBTDL before and after the relaxation process.

The materials, after relaxation, were examined by FTIR-ATR and Raman spectroscopies to confirm that no structural effects had occurred after heating the sample to 195 °C. Although the reversible reaction mechanism behind the relaxation process was determined to be dissociative, we could not detect the presence of isocyanate or thiol in the spectra, since the coupling reaction of isocyanate and thiol in these conditions is extremely fast. Figure 9 shows the FTIR spectra of the material before and after the relaxation process.

As observed in the figure, the chemical structure of the material after relaxation process in DMTA is maintained unaltered, with the main peak of the C=O of thiourethane groups at 1670 cm^−1^. No isocyanate absorption at 2250 cm^−1^ has been formed. By Raman spectroscopy, we could not detect the -SH absorption around 2600 cm^−1^, which, in this spectroscopy, appears as a strong band, whereas in the FTIR it is difficult to see due to its weak intensity. No differences could be observed between acid- and base-catalyzed samples.

### 3.5. Self-Healing Behavior

**Figure 10:**
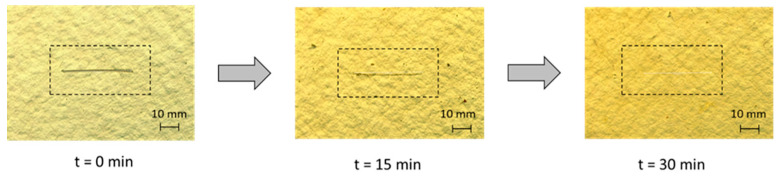
Optical microscope images of the thermal self-healing process at 190 °C at different times for sample SQLM 2575 BASE.

Due to the presence of thiourethane groups, which are able to establish hydrogen bonding and to rearrange by trans thiocarbamoylation reaction, it could be foreseen that the materials reported in this work can be self-healed effectively at an adequate temperature. Both covalent and non-covalent bonds will break during the damage event, but both can be formed again by different mechanisms. Usually, only one of these covalent or non-covalent mechanisms is involved; therefore, the concurrence of both can act in a cooperative way. Temperature will be, in this case, the external trigger [28]. The double dynamic repairing mechanism was firstly proposed by Lehn and colleagues based on the rearrangement of bis-acylhydrazones (reversible covalent links) and hydrogen bonding interactions (non-covalent linkages) [29]. Figure 10 shows some optical microscope images of the samples captured during the self-healing process.

As can be seen, the scratch disappeared in only 30 min, which confirms the good ability of this material (it corresponds to the sample that relaxed faster) to be self-healed in a short time. It is important to highlight that the chemistry involved in the preparation of these materials is more simple than other typical self-healing systems involving Diels-Alder adducts [30] or S-S disulfide exchange [31].

**Figure 11:**
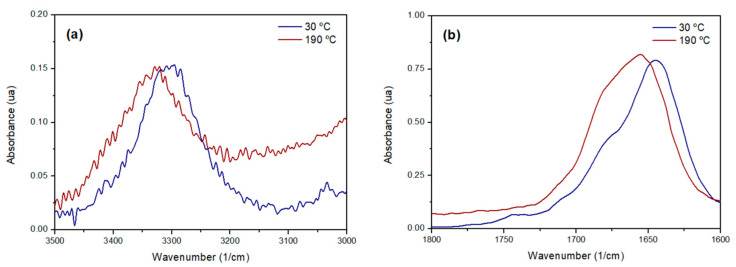
FTIR-ATR spectra of the sample SQLM 2575 BASE expanded in the regions of N-H (**a**) and C=O (**b**) vibrations at 30 and 190 °C temperatures.

To demonstrate that the hydrogen bonding between thiourethane groups can be involved in the self-healing process, FTIR spectra of the sample SQLM 2575 BASE have been recorded at 30 °C and 190 °C. As seen in Figure 11, the temperature change significantly affects the FTIR bands related to the hydrogen bonds. On increasing the temperature, the stretching bands of N-H and C=O bonds displace to higher wavenumber, indicating the breakage of these non-covalent interactions [32]. This process is fully reversible. Thus, these bonds’ breakage and subsequent formation can help the material to self-heal in a cooperative way.

## 4. Conclusions

A series of biobased polythiourethanes with vitrimer-like characteristics has been prepared from hexamethylene diisocyanate and two renewable thiols derived from squalene and limonene, which were previously synthesized. Different proportions of both thiols: 25/75, 50/50, and 75/25 were tested. The catalyst BGDBU, a salt that releases the base upon heating, and DBTDL, as a Lewis acid, were used.

The materials obtained had *T_g_*s between 86 and 107 °C, which increased with an increase in the amount of the squalene derivative in the formulation due to the tighter network structure.

The thermal stability of these materials allows their safe manipulation up to temperatures higher than 200 °C.

The fastest relaxation was observed for the materials with the lowest ratio of squalene thiol. Moreover, the materials prepared with a basic catalyst relax much faster than those prepared with DBTDL. Thus, a relaxation time (τ_0.37_) as short as 10 s was determined for the SQLM 2575 sample catalyzed by BGDBU. The relaxation process is based on the trans thiocarbamoylation process, which was previously reported. This process leads to vitrimer-like characteristics in the relaxation of the materials since it follows an Arrhenius-type dependence of the relaxation time on the temperature.

The topology freezing temperatures (*T_v_*s) are higher than the *T_g_*s, which implies that the materials have to exceed both temperatures to be reshaped.

The self-healing test demonstrates that the sample SQLM 2575 BASE can be completely healed after 30 min at 190 °C. This can be attributed to the presence of exchangeable thiourethane covalent bonds and hydrogen bond interactions in the network structure.

## Figures and Tables

**Table 1:** Composition and nomenclature of the samples of renewable thiols prepared.

Sample	HDI (g)	SQ-S6 (g)	LM-S2 (g)	Catalyst (g)
SQLM 2575 ACID	1.27	0.39	1.16	0.19
SQLM 5050 ACID	1.27	0.77	0.77	0.19
SQLM 7525 ACID	1.27	1.16	0.39	0.19
SQLM 2575 BASE	1.29	0.39	1.18	0.14
SQLM 5050 BASE	1.29	0.78	0.78	0.14
SQLM 7525 BASE	1.29	1.18	0.39	0.14

**Table 2:** Main thermal data obtained from TGA tests in nitrogen atmosphere.

Material	*T*_5%_ ^a^ (°C)	*Residue* ^b^ (%)	*T_peak_*_1_ ^c^ (°C)	*T_peak_*_2_ ^c^ (°C)
SQLM 2575 ACID	258	2.00	308	360
SQLM 5050 ACID	264	1.63	308	363
SQLM 7525 ACID	268	1.13	306	365
SQLM 2575 BASE	248	1.16	308	356
SQLM 5050 BASE	251	1.04	308	359
SQLM 7525 BASE	252	0.70	301	365

^a^ Temperature of 5% of weight loss, ^b^ Char residue at 700 °C, ^c^ Temperature of the maximum rate of degradation of two main steps.

**Table 3:** Main data obtained from DMTA analysis of the materials prepared.

Material	*T*_tan__*δ*_ ^a^ (°C)	*FWHM* ^b^ (°C)	*E’_glassy_* ^c^ (MPa)	*E’_rubbery_* ^d^ (MPa)
SQLM 2575 ACID	86	16	1761	2
SQLM 5050 ACID	95	17	1817	5
SQLM 7525 ACID	107	18	1843	15
SQLM 2575 BASE	91	15	1802	3
SQLM 5050 BASE	101	16	1877	9
SQLM 7525 BASE	107	19	1893	17

^a^ Temperature at the maximum of the tan *δ* peak at 1 Hz. ^b^ Full width at half maximum of the tan *δ* peak. ^c^ Glassy storage modulus determined by DMTA at *T_g_* −50 °C. ^d^ Rubbery storage modulus determined by DMTA at *T_g_* +50 °C.

**Table 4:** Main data extracted from relaxation-stress experiments of the materials.

Material	*τ*_0.37_ ^a^ (s)	*E_a_* (kJ/mol)	ln *A* (s)	*r^2^*	*T_v_* ^b^ (°C)
SQLM 2575 ACID	165	118	26.4	0.9801	104
SQLM 5050 ACID	342	138	30.7	0.9833	120
SQLM 7525 ACID	2232	144	31.4	0.9996	140
SQLM 2575 BASE	10	105	21.7	0.9675	108
SQLM 5050 BASE	36	117	23.4	0.9573	132
SQLM 7525 BASE	190	127	24.8	0.9529	150

^a^ Time to reach a value relative of *σ*/*σ_o_* = 0.37 at 180 °C. ^b^ Topology freezing temperature.

**Table 5:** Main data extracted from creep experiments of the materials catalyzed by BGDBU.

Material	*E_a_* (kJ/mol)	*r* ^2^	*T_v_* ^a^ (°C)	Fragility Index ^b^ (m)
SQLM 2575 BASE	97	0.9675	143	13.9
SQLM 5050 BASE	111	0.9573	154	15.5
SQLM 7525 BASE	135	0.9529	160	18.6

^a^ Topology freezing temperature obtained from creep tests. ^b^ The Fragility index for Silica is 16.5.

## Data Availability

The data presented in this study are available on request from the corresponding author. The data are not publicly available due to the policy of protection in our research team.
